# Retrospective Analysis of Suspected Rabies Cases Reported at Bugando Referral Hospital, Mwanza, Tanzania

**DOI:** 10.4103/0974-777X.68530

**Published:** 2010

**Authors:** Humphrey D Mazigo, Fredros O Okumu, Eliningaya J Kweka, Ladslaus L Mnyone

**Affiliations:** *Department of Medical Parasitology and Entomology, Weill-Bugando University of Health Sciences, P.O. Box 1464, Mwanza, Tanzania*; 1 *Biomedical and Environmental Group, Ifakara Health Institute, P.O. Box 53, Ifakara, Tanzania*; 2 *Division of Livestock and Human Disease Vector Control, Mosquito Section, Tropical Pesticide Research Institute, Box 3024, Arusha, Tanzania*; 3 *Pest Management Centre, Sokoine University of Agriculture P.O. Box 3110, Morogoro, Tanzania*

**Keywords:** Bugando hospital, Human animal bites, Mwanza, Rabies, Tanzania

## Abstract

**Aims:**

The aim of this study was to determine the incidence of humans being bitten by rabies-suspected animals, and the victims’ adherence to post-exposure prophylaxis (PEP) regimen.

**Materials and Methods:**

A retrospective analysis of data of victims treated at Bugando Medical Centre during the period 2002-2006 (*n*=5 years) was done.

**Results:**

A total of 767 bite injuries inflicted by rabies-suspected animals were reported, giving a mean annual incidence of ~58 cases per 100,000 (52.5% males, 47.5% females). The proportion of children bitten was relatively higher than that of adults. All victims were treated by using inactivated diploid-cell rabies vaccine and were recommended to appear for the second and third doses. However, only 28% of the victims completed the vaccination regime. Domestic dogs were involved in 95.44% of the human bite cases, whereas cats (3.9%), spotted hyena (*Crocuta crocuta*) (0.03%), vervet monkey (*Cercopithecur aethiops*) (0.01%) and black-backed jackal (0.01%) played a minor role. The majority of rabies-suspected case reports were from Nyamagana district and occurred most frequently from June to October each year.

**Conclusions:**

In conclusion, this study revealed that incidences of humans being bitten by dogs suspected of rabies are common in Tanzania, involve mostly children, and victims do not comply with the prophylactic regimen. Rigorous surveillance to determine the status of rabies and the risk factors for human rabies, as well as formulation and institution of appropriate rabies-control policies, is required.

## INTRODUCTION

Rabies is a highly fatal, zoonotic disease that causes severe destruction of the central nervous system of all warm-blooded animals. Typically, humans acquire rabies following the bite of a rabid animal. Domestic dogs (*Canis familiaris*) play a key role in the transmission of rabies to humans. About 85% to 95% of human rabies cases in China; and 94% to 98%, in India and Pakistan were ascribed to dog bites.[1] In the United Republic of Tanzania, 23,709 humans sustained dog-bite injuries between 1990 and 1996,[2] whereas 42,669 human dog-bite injuries were reported for the year 2000 by Cleaveland and others,[2] suggesting that there was either an increase in the number of cases or the extent of the problem was being underestimated in the reports.

The vast majority (99%) of human deaths arising from rabies occur in the tropical developing world.[3,4] About 24,000 to 70,000 people are estimated to die of rabies each year in Africa and Asia.[5] Most of the incidences of human rabies occur in rural areas. It has been proposed that this is due to a number of reasons, including (i) low vaccination coverage of dogs as a result of inadequate awareness of the problem, as well as inability to finance the costs of vaccination; (ii) poor management of dogs, in particular the free movement of dogs, which increases their risk of contracting rabies from wildlife;[6] and (iii) although effective and economical control measures are available,[7,8] rabies remains a neglected disease in terms of policy formulations throughout most of the developing countries.[4,9] The lack or low level of political commitment to control rabies is perhaps due to lack of accurate data about rabies to clearly show its impact on public health and socioeconomic affairs.

The human population boom in Africa appears to correlate well with the increase in the number of domestic dogs.[10] Nevertheless, by conservative estimates, the prevalence of human rabies is considered to be under-reported.[3] The under-reporting is largely attributed to poor surveillance systems and/ or people’s tendency not to report human and animal cases of rabies. Sole reliance on clinical diagnosis (in animals and/ or humans) is also downgrading the reliability of rabies-surveillance systems. Thus there has been poor planning resulting in poor availability and inappropriate administration of post-exposure prophylaxis (PEP), as well as delays in its administration.[11] Although not as frequent as the inability to afford treatment, such scenarios have built a lack of trust in health facilities, as well as poor compliance with PEP regimens. Other reasons given for not reporting, completing or adhering to PEP include poor awareness about the danger of the disease, small size of the injury, reluctance of the dog owner to pay for treatment costs, and not being advised to take PEP.[11]

If used keenly, reports of animal-bite injuries would aid in estimating area- or region-specific disease burden, thus enabling giving priority to improved rabies surveillance and control. Reports of animal-bite victims in the hospitals and health centers are examples of resources for such information. Such reports would assist in identifying (i) characteristics of patients reporting (age, sex); (ii) areas with frequent incidences of animal bites; (iii) species of animals involved; and (iv) periods with high incidence of bite injuries. These insights would prompt surveillance providers to better understand how dog population size, movements, accessibility and habitat affect the transmission of rabies. Furthermore, it would be helpful to understand people’s level of awareness of the disease, and the availability and accessibility of practical rabies-control strategies. Therefore, the aim of this study was to retrospectively study records of animal-bite incidences at Bugando Medical Centre, Mwanza, Tanzania, from 2002 to 2006.

## MATERIALS AND METHODS

Data were collected from the hospital records of humans who were bitten by animals suspected to have rabies for the period of five years from 2002 until 2006. The information gathered included date of attendance, sex, age, geographical location (district and village) of the patient, species of the animal that bit them and compliance to the vaccinations.

### Data analysis

Data was firstly aggregated by districts, years, age and sex. The number of cases was converted into incidences (bites/100,000) based on population size projected from the 2002 census counts and estimated population-growth rates (http://www.tanzania.go.tz/census/regions.htm). Incidences and percentages were calculated in Microsoft™ Excel.

### Ethical issues

The study was approved by a joint committee of Research Ethics and Publication of Bugando Medical Hospital and Weill-Bugando University College of Health Sciences, Mwanza, Tanzania.

## RESULTS

**Figure 1:**
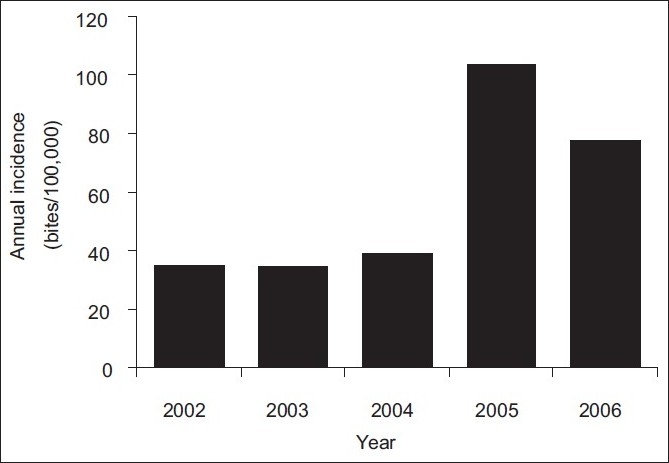
Total annual incidence of cases of humans being bitten by animals during the five-year period from 2002 to 2006

**Figure 2:**
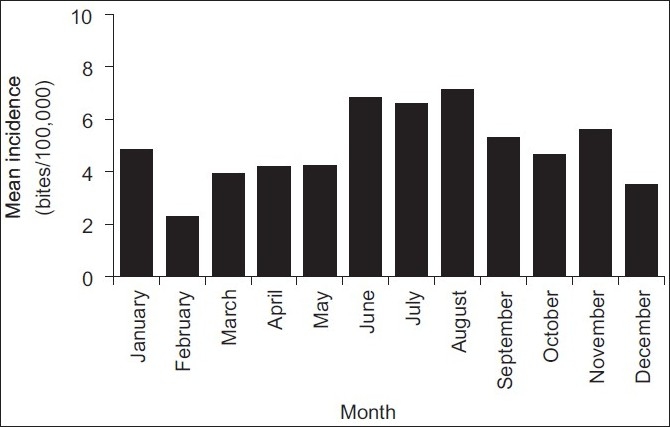
Mean monthly incidence of cases of humans being bitten by animals recorded across the five-year period from 2002 to 2006

During the 5-year period (2002-2006), a total of 767 bite injuries by rabies-suspected animals were reported at Bugando Hospital, giving a mean annual incidence of ~58 cases per 100,000 (52.5% males, 47.5% females). Children (<18 years of age) constituted 55% of the total number of cases. Domestic dogs constituted the highest proportion (95.44%) of animals suspected to have rabies that bit humans. Other animals included cats (3.9%), spotted hyena (*Crocuta crocuta*) (0.03%), vervet monkey (*Cercopithecur aethiops*) (0.01%) and black-backed jackal (0.01%). The number of cases increased through time, with the highest number of patients, viz., 275, being recorded in 2005 [Figure 1, incidence of 103.4 cases per 100,000]. Interestingly, each year, the majority of cases were recorded during the period June to October [Figure 2].

**Figure 3:**
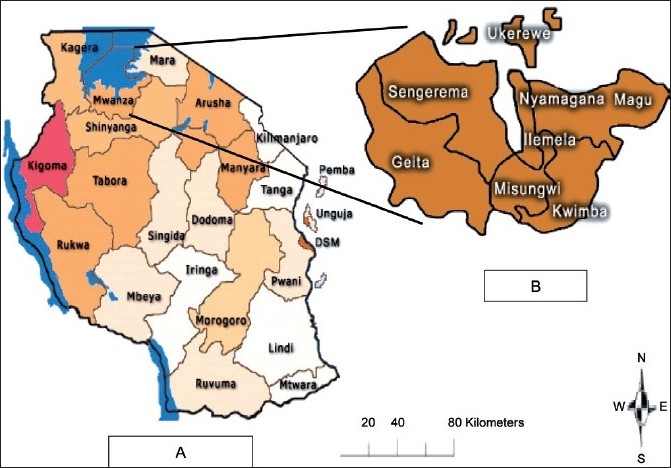
A map showing a) Mwanza in relation to other regions of Tanzania and b) districts of Mwanza region

**Figure 4:**
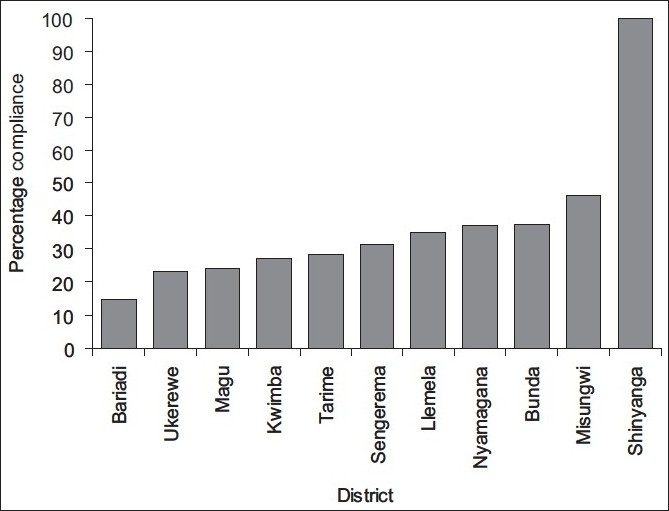
Percentage compliance by animal-bite victims to all the three recommended doses of human rabies vaccination in the five-year period from 2002 to 2006

As expected, the majority (92.05%) of victims were from Mwanza region. The rest (7.95%) came from the nearby regions, including Tabora, Shinyanga and Mara [Figure 3]. Of the 767 cases, 471 victims hailed from Nyamagana district, giving mean annual incidence of 41.93 cases per 100,000. Few other cases were reported from other districts within and without Mwanza region [Table 1]. There was no confirmed human rabies case during the entire reporting period. All patients who reported having been bitten by rabies-suspected animals were vaccinated with the human diploid-cell vaccine (H DCV) (Rabivax®) inactivated by using propiolactone. Although the recommendation in Tanzania at Bugando Hospital is that the anti-rabies vaccine be administered in three doses of 1 mL each on days 0, 3 and 14, the majority (71.9%) of patients did not return to hospital to complete the follow-up vaccinations. The average compliance for completion of the vaccination regime was 28.09%. The rate of compliance varied between locations. The highest (100%) compliance was recorded for victims from Shinyanga district in Shinyanga region [Figure 4]. Out of 471 victims from Nyamagana, only 175 (37.15%) complied with the complete vaccination regime.

**Table 1:** Total number of cases of humans being bitten by animals according to district recorded across the five-year period from 2002 to 2006

Region	District	No. of victims
Mwanza	Nyamagana	471
	Misungwi	67
	Magu	58
	Ilemela	37
	Kwimba	22
	Ukerewe	25
	Sengerema	20
	Geita	6
Shinyanga	Shinyanga urban	11
	Kahama	5
	Bariadi	22
Mara	Tarime	7
	Bunda	8
	Musoma urban	1
	Serengeti	1
Kagera	Biharamulo	4
Tabora	Tabora urban	2
Total		767

## DISCUSSION

Children constituted the highest proportion of human cases reported to Bugando Referral Hospital for treatment of injuries caused by bites of rabies-suspected animals, mainly domestic dogs. Many of the reported victims were residents of rural areas. Case-reporting at Bugando may be biased depending on the proximity to the hospital; nonetheless, we have observed that distribution according to locality and age of the patient still corroborates with reports from elsewhere.[2,12,13]

Among bites by various animals, dog bites being the major cause of injury to humans is supported by previous findings in many parts of the world, particularly Africa and Asia.[1,6] Domestic dogs are the principal reservoirs and vectors of rabies.[14,15] Therefore, the increased number of humans bitten by, or exposed to, rabies-suspected dogs observed in this study reflects the increased threat of rabies transmission. It should be emphasized that, obviously, a mean annual incidence of ~58/100,000 human animal bites is much less than the actual number of animal-bite cases, since many people would not normally report to the hospitals unless they suspect or are certain about abnormal behaviors of dogs, which is the case most frequent in the rural areas of Tanzania, and elsewhere; and many people may not be able to receive treatment or recognize this as a requirement. Furthermore, the number of dog bites is likely to increase with increase in human population and the increased usage of dogs as companions and guard animals. Consequently, there will likely be a substantial increase in rabies transmissions and associated deaths, especially in the rural and poverty-stricken areas, where dogs are not leashed, have free movement, thereby increasing the risk of exposure to rabies.[6] Understanding the dynamics of rabies in domestic dogs is needed to facilitate the designing of more effective control measures that could reduce deaths from the disease, of which >95% result from bites by domestic dogs.[6,14]

Human bites by species other than dogs were also reported. The species included domestic cats and wild canids, namely, spotted hyena, vervet monkey and black-backed jackal. Although there is evidence that some wild canid populations in Africa can support rabies cycles,[16] most outbreaks in the wild canids are triggered by epidemics in domestic dogs rather than the converse.[11,17,18]

Given the reported endemicity of rabies in Africa, including Tanzania,[8] all victims who presented to the hospital with bites from rabies-suspected animals were considered for vaccination. However, only 28% of the victims who received the first dose completed the two subsequent doses. This finding is in agreement with previous reports, that exposed people do not receive a full course of treatment and often delay starting prophylactic vaccination treatment.[19,20] The observation that a large percentage of victims do not complete the mandatory PEP, irrespective of the increased risk of dog bites, suggests more transmission given the endemicity of rabies in Tanzania. Our above finding highlights a serious problem and indicates the need to educate the victims and public on the risk and danger of the disease.

Most of the victims came from Nyamagana district, where Bugando Hospital is located. This proximity may have contributed to an increased number of victims reporting from Nyamagana. Other districts within and without Mwanza region have district hospitals, where people from those areas may have been reporting rather than coming to Bugando. People from other districts could have reported at Bugando in case of shortages in their hospitals and/ or getting bite injuries while visiting relatives.

The increasing trend in the number of reported cases in recent years, with the highest incidence of cases reported in 2005 (103.4/100,000), can be explained by several factors: i) heightened awareness about the consequences of rabies; ii) increased availability of modern post-exposure treatment (PET) and enhanced access to medical facilities for PET, thus increasing people’s trust in services and tendency to report; iii) increased dog population and movements; and iii) poor adherence to rabies-control measures for dogs, such as dog vaccination and population control, leading to many bites and more reports of such cases. The year 2005 was also a year with the most dog bites in Mara region, indicating a large-scale epidemic in those regions.[11] Such epidemic may have also contributed to the increased frequency of reporting shown in the present study. An increasing rabies incidence in Africa and Asia has been largely attributed to population growth of dogs.[6,21]

In Tanzania, anti-rabies dog vaccine is available in most of the veterinary centers. The World Health Organization (WHO) estimates the threshold vaccination coverage for rabies eradication in dog populations at about 70% based on empirical evidence.[22] Rabies vaccination campaigns in rural Africa have resulted in reductions in cases of rabies in dog populations by 70%[8] However, as indicated earlier, our data suggests that cases of humans being bitten by rabies-suspected dogs are on the rise. Clearly, there is need for increased vaccination coverage of dogs to reduce rabies transmission among the susceptible animals and humans. Dog vaccination campaigns would even be more successful when accompanied with accessible and affordable PEP for humans. A number of constraints to achieving adequate coverage through mass dog vaccination include poor infrastructure, lack of finance, poor awareness and thus insufficient community participation; however, with appropriate efforts, these can all be addressed.

## CONCLUSIONS AND RECOMMENDATIONS

This study revealed poor compliance of dog-bite victims to post-exposure prophylaxis, irrespective of the increased risks of contracting the disease and death, given the rise in the number of dog bites and the endemicity of rabies in Tanzania. Therefore, the study suggests a need for deliberate efforts to educate the public about the risks of rabies. Also, since domestic dogs are the major transmitting agent of rabies to humans, it is vital to better understand how their population size, age/ sex structure, movements, accessibility and habitat affect rabies spread in Tanzania. Such information would aid in the development of improved control strategies.
